# Metabolomic Fingerprinting of *Salinispora* From Atlantic Oceanic Islands

**DOI:** 10.3389/fmicb.2018.03021

**Published:** 2018-12-11

**Authors:** Anelize Bauermeister, Karen Velasco-Alzate, Tiago Dias, Helena Macedo, Elthon G. Ferreira, Paula C. Jimenez, Tito M. C. Lotufo, Norberto P. Lopes, Susana P. Gaudêncio, Letícia V. Costa-Lotufo

**Affiliations:** ^1^Departamento de Farmacologia, Instituto de Ciências Biomédicas, Universidade de São Paulo, São Paulo, Brazil; ^2^Núcleo de Pesquisa em Produtos Naturais e Sintéticos (NPPNS), Faculdade de Ciências Farmacêuticas de Ribeirão Preto, Universidade de São Paulo, São Paulo, Brazil; ^3^UCIBIO-REQUIMTE, Departamento de Química, Laboratório de Biotecnologia Azul e Biomedicina, Faculdade de Ciências e Tecnologia, Universidade NOVA de Lisboa, Caparica, Portugal; ^4^UCIBIO-REQUIMTE, Departamento de Ciências da Vida, Laboratório de Biotecnologia Azul e Biomedicina, Faculdade de Ciências e Tecnologia, Universidade NOVA de Lisboa, Caparica, Portugal; ^5^Departamento de Química Orgânica e Inorgânica, Universidade Federal do Ceará, Fortaleza, Brazil; ^6^Departamento de Ciências do Mar, Universidade Federal de São Paulo, São Paulo, Brazil; ^7^Instituto Oceanográfico, Universidade de São Paulo, São Paulo, Brazil

**Keywords:** *Salinispora*, saliniketal, metabolomic, molecular networking, LC-MS

## Abstract

*Salinispora* (Micromonosporaceae) is an obligate marine bacterium genus consisting of three species that share over 99% 16S rRNA identity. The genome and biosynthetic pathways of the members of this genus have been widely investigated due to their production of species-specific metabolites. However, despite the species’ high genetic similarity, site-specific secondary metabolic gene clusters have been found in *Salinispora* strains collected at different locations. Therefore, exploring the metabolic expression of *Salinispora* recovered from different sites may furnish insights into their environmental adaptation or their chemical communication and, further, may lead to the discovery of new natural products. We describe the first occurrence of *Salinispora* strains in sediments from the Saint Peter and Saint Paul Archipelago (a collection of islets in Brazil) in the Atlantic Ocean, and we investigate the metabolic profiles of these strains by employing mass-spectrometry-based metabolomic approaches, including molecular networking from the Global Natural Products Social Molecular Networking platform. Furthermore, we analyze data from *Salinispora* strains recovered from sediments from the Madeira Archipelago (Portugal, Macaronesia) in order to provide a wider metabolomic investigation of *Salinispora* strains from the Atlantic Oceanic islands. Overall, our study evidences a broader geographic influence on the secondary metabolism of *Salinispora* than was previously proposed. Still, some biosynthetic gene clusters, such as those corresponding to typical chemical signatures of *S. arenicola*, like saliniketals and rifamycins, are highly conserved among the assessed strains.

## Introduction

*Salinispora* is a genus of actinobacteria that has been widely studied and considered as a prolific source of natural products due to its unique biosynthetic pathways, which lead to the production of rich and diverse structures of secondary metabolites (Jensen et al., 2015). The genus belongs to the family Micromonosporaceae and comprises three distinct and closely related species, *S. arenicola, S. pacifica*, and *S. tropica*, which share 99% 16S rRNA gene sequence identity (Maldonado et al., 2005; Jensen and Mafnas, 2006). However, recent phylogenetic studies based on full genome sequencing suggested the existence of 10 candidate *Salinispora* species (Millan-Aguinaga et al., 2017).

**FIGURE 1:**
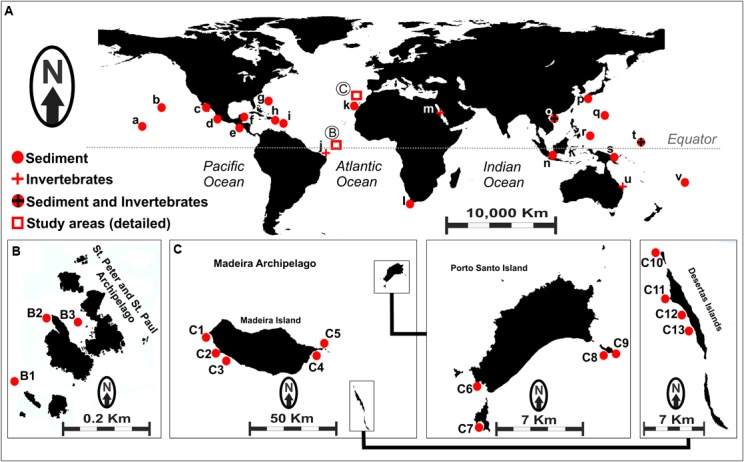
**(A)** Map showing worldwide occurrence of *Salinispora* genus, based on previously reported data. (a) Palmyra; (b) Hawaii; (c) Gulf of California; (d) Mexico (Pacific Coast); (e) Costa Rica; (f) Mexico (Caribbean); (g) United States Virgin Islands; (h) Bahamas; (i) Dominica; (j) Rocas Atoll (unpublished); (k) Canary Islands; (l) South Africa; (m) Red Sea; (n) Malaysia; (o) South China Sea; (p) Japan; (q) Guam; (r) Palau; (s) Papua New Guinea; (t) Micronesia; (u) Great Barrier Reef; (v) Fiji. Circles represent sediment-recovered strains, and crosses represent invertebrate-recovered strains. **(B)** Sampling sites (B1–B3) in the Saint Peter and Saint Paul Archipelago, Brazil. **(C)** Sampling sites (C1–C13) in the Madeira Archipelago, Portugal. Details for the samples obtained from each location assessed in this study are shown in Supplementary Table S1.

Although *S. tropica* has a restricted geographic distribution and has been found only in the Bahamas and Yucatán, *S. arenicola* and *S. pacifica* are widely distributed in tropical and subtropical oceans (Figure 1A; Bose et al., 2014; Goo et al., 2014; Millan-Aguinaga et al., 2017). The genus was found in several locations in the Pacific Ocean, whereas the only report of cultured *Salinispora* genus in the Atlantic Ocean is from sediment samples from the Madeira Archipelago (Prieto-Davo et al., 2016). It is worth noting that the authors of the latter study recovered many *S. pacifica* strains, including four novel phylotypes, but only one *S. arenicola* strain. Nevertheless, Ferreira et al. (2016) reported evidence of the occurrence of *Salinispora* in sediments from the Saint Peter and Saint Paul Archipelago, Brazil, based on the identification of metabolites produced by marine actinobacteria that have been associated specifically with *Salinispora* species.

Despite the high genetic similarity of *Salinispora* species, the production of some secondary metabolites is species-specific; for example, saliniketal and rifamycin are produced only by *S. arenicola*, whereas salinispyrone is produced only by *S. pacifica* and salinisporamide only by *S. tropica* (Jensen et al., 2007, 2015). In addition, environmental settings seem to play a key role in microorganisms’ metabolite production (Louca et al., 2016; Costa-Lotufo et al., 2018) and in the evolution of the genus *Salinispora*, because certain secondary metabolic gene clusters are endemic to specific locations (Edlund et al., 2011; Letzel et al., 2017). Therefore, assessing the metabolomic profile of this actinobacteria from different locations and distinct environmental conditions may facilitate insights into the relation between this chemically prolific genus and the environment.

Mass spectrometry (MS) strategies are important tools for investigating the chemical diversity in biological systems, because they enable the rapid analysis of a large range of chemical classes in complex samples (Aksenov et al., 2017). Furthermore, fragmentation reactions using MS can provide important structural information, facilitating the identification of compounds present in these samples (Bauermeister et al., 2016; Demarque et al., 2016). In this context, MS-based metabolomic approaches have enabled the investigation of genome expression by analyzing the metabolite content of an organism in a defined condition. Such studies have been successfully used to obtain information regarding evolution in an ecological context and to assess phylogenetic relationships (Ernst et al., 2015; Brunetti et al., 2018). Therefore, MS-based metabolomics have been widely employed in the investigation of microbial metabolite content owing especially to its high sensitivity, which allows for the detection of molecules even at low concentrations in complex samples. Considering that similar chemical structures might present a similar fragmentation pattern, molecular networking, an online molecular tool at Global Natural Products Social Molecular Networking (GNPS) (Wang et al., 2016), enables the clustering of such related chemical structures based on their spectral similarities. By comparison with a library of known molecules, this tool helps to accelerate the dereplication process (Gaudencio and Pereira, 2015) of complex samples such as microbial crude extracts without arduous data mining.

In this study, we describe the recovery of bacteria from the genus *Salinispora* from sediments of the Saint Peter and Saint Paul Archipelago (Brazil), in the equatorial Atlantic Ocean. Therefore, in order to investigate the metabolites produced by *Salinispora* strains reported here, we employed metabolomic tools, such as molecular networking from GNPS platform. Furthermore, extracts produced by the *Salinispora* strains recovered from the Madeira Archipelago (Portugal) were added to the analyses in order to expand and compare the metabolic expression of *Salinispora* from different locations in the Atlantic Ocean. By conducting a broader assessment of the chemical diversity hosted by certain species, including that of specimens from different geographic locations throughout the Atlantic, an undersampled ocean, this study offers a fuller understanding of the relationships between *Salinispora* actinobacteria and the environment, and it expands the scientific awareness of the already prolific secondary metabolism of this group of microorganisms.

## Materials and Methods

### Bacteria Isolation

A total of 54 actinobacteria strains were used in the present study, including 20 strains recovered from sediments collected off the Saint Peter and Saint Paul Archipelago – Brazil (codes BRA), as described by Ferreira et al. (2016), and 34 strains recovered from sediment samples collected off the Madeira Archipelago – Portugal (codes PTM), as described by Prieto-Davo et al. (2016). Sampling sites are shown in Figure 1B (Brazil) and Figure 1C (Portugal), and other details regarding the sediment samples are given in Supplementary Table S1.

### Crude Extract Preparation

BRA and PTM *Salinispora* strains were cultured in Erlenmeyer flasks containing A1 media following the procedures of Ferreira et al. (2016) and Prieto-Davo et al. (2016), respectively. For BRA strains, 1 mL aliquot of each bacterial culture was separated for genomic DNA extraction (Garcia et al., 2013). The liquid cultures were extracted with ethyl acetate, and the organic phase was dried under reduced pressure and kept at 4°C. For metabolomic analyses, all crude extracts were diluted in methanol at 1.0 mg/mL.

### DNA Extraction, 16S rRNA Amplification and Sequencing

Of each BRA culture broth, 1 ml was centrifuged (2,000 g/3 min), washed with CTAB buffer, and treated with 1.4 M NaCl, 20 mM EDTA, 100 mM Tris-HCl (pH 8.0), 5 μg/mL proteinase K, and 0.5% (v/v) of 2-mercaptoethanol. Samples were then frozen at -80°C for 3–5 min and thawed in a dry bath for 3 min at 65°C (this process was repeated twice). Next, an equal volume of a solution containing phenol/chloroform/isoamyl alcohol 25:24:1 was added to each tube, homogenized by inversion, and centrifuged (7 min, 14,000 *g*, 4°C). The aqueous phase was recovered, mixed with 400 μL chloroform, and centrifuged (5 min, 14,000 *g*, 4°C). Genomic DNA was precipitated with ammonium acetate and cold isopropyl alcohol and subsequently centrifuged. The supernatant was discarded, and the pellet formed was washed with cold ethanol (70%), centrifuged, and solubilized in TE buffer (10 mM Tris-HCl and 1 mM EDTA). Genomic DNA was quantified using NanoDrop^TM^. To assess the integrity and purity of the DNA, an agarose gel (1%) was stained with ethidium bromide and visualized in a transilluminator.

The 16S rRNA gene for Brazilian samples was amplified by polymerase chain reaction (PCR) using the same primers previously used for Portuguese strains by Prieto-Davo et al. (2016), 27F (5′-AGAGTTTGATCCTGGCTCAG-3′) and 1492R (5′-TACGGCTACCTTGTTACGACTT-3′) (Gontang et al., 2007). The PCR mix contained PCR buffer, DNTPs, MgCl_2_, and Taq polymerase. The protocol conditions included an initial denaturation period at 94°C, followed by 35 cycles of denaturation at 94°C for 60 s, annealing phase under 63°C for 60 s, and extension at 72°C for 60 s. The cycling phase, a final extension at 72°C for 10 min, was subsequently performed. The PCR products were visualized in a 1% agarose gel electrophoresis and stained with SYBR^TM^. The stained DNA band was cut from the gel and purified by Ilustra^TM^ GFX^TM^ PCR DNA and a Gel Band Purification Kit (GE Healthcare Life Sciences). The sequencing of the 16S rRNA gene was performed by Macrogen Inc. (Seoul, Korea) with the ABI PRISM BigDye^TM^ Terminator cycle sequencing kit (Applied Biosystems, United States), and the same primers were used for amplification, following the protocols provided by the manufacturer. Raw 16S rRNA sequence data for Portuguese samples (PTM) were kindly provided by Dr. Susana Gaudêncio and were obtained as described by Prieto-Davo et al. (2016).

### Phylogenetic Analysis

Using Geneious 7 software (Biomatters Ltd.), the forward and reverse sequences from both BRA and PTM strains were aligned and the consensus sequences were obtained. All the sequences from BRA and PTM strains reported in this study were deposited in GenBank under accession numbers MH910676 through MH910695 and KT446218 through KT446251, respectively^1^. Sequences were compared to the EzBioCloud database^2^ and then aligned with MAFFT version 7 (Katoh and Standley, 2013). For the phylogenetic reconstruction, the general time reversible nucleotide substitution model with gamma distribution was selected using jmodeltest2 (Darriba et al., 2012). The phylogenetic relationships were inferred through a maximum likelihood approach using RaxML (Stamatakis, 2006) with 1,000 bootstrap iterations. Sequences of three different *Micromonospora* lineages were used as an outgroup to root the tree (*M. pattaloongensis* MM 60 T, *M. echinofusca* DSM 43913 T, and *M. inyonensis* DSM 46123 T, accession numbers JN54581.1, NR_044891-1, and NR_044893.1, respectively). Other *Salinispora* sequences from candidate species from the work of Millan-Aguinaga et al. (2017) were obtained from Integrated Microbial Genomes and Microbiomes^3^ and included in order to confirm the species’ identities and compare the genus diversity. All codes of the used sequences were included in the tree. The tree was visualized and edited in Interactive Tree of Life version 3 (Letunic and Bork, 2016).

### Mass Spectral Data Acquisition

For metabolomic content evaluation, all crude extracts were evaluated by HPLC-MS/MS. The analyses were developed on an HPLC system (Shimadzu) coupled to an AmaZon SL mass spectrometer (Bruker Daltonics) fitted with an electrospray ionization source operating in positive ionization mode and with an ion trap MS detector. The chromatographic separation occurred on a Phenomenex Luna C18(2) (5 μm, 250 mm × 4.6 mm) column, using a gradient from 5 to 100% MeOH over 35 min followed by 100% MeOH for 7 min, with a flow rate of 1.0 mL/min. Both solvents contained 0.1% formic acid. The column temperature was set to 40°C. (Ion source: ESI, voltage: 3,500 V, capillary temp.: 310 °C, *m/z* range: 100–1,200, gas pressure: 40 psi.) An untargeted method was employed for acquisition of MS/MS spectra, in which the analyzer selects in each MS scan the three highest-intensity ions to fragment, using a ramp of collision energy from 20 to 75 eV.

### Data Processing and Multivariate Analysis

HPLC-MS data, containing the total ion chromatogram along with retention time peaks and their corresponding mass spectra, were exported to the software MZmine 2.32 (MZmine VTT, Finland) for data processing. For peak detection, it was considered the signal-to-noise ratio higher than 5.0E6 using the exact mass algorithm. The chromatogram builder function was set at 0.1 min as the minimum time span and 6.0E6 as the minimum height. Chromatogram deconvolution was performed using the local minimum search algorithm (minimum relative height = 10%, minimum absolute height = 6.0E6, minimum ratio of peak top/edge = 1.2). Isotopes from the same compound were grouped, and the data were then filtered to remove duplicate peaks. The resulting peak list was aligned using the join aligner method. Finally, adduct searching was performed to discard different ions from the same compound. The resulting .csv table was subjected to Pareto scaling. This table was used to generate principal component analysis (PCA), hierarchical cluster analysis (HCA), and a heatmap using the software R (version 3.3.1, 64-bit) (R Foundation for Statistical Computing, Vienna, Austria) with a script employing factoextra, dendextend, and g-plot packages, respectively. HCA was performed using Euclidian distance and amalgamated by Ward’s method.

### Molecular Networking

HPLC-MS/MS raw data files were converted from .raw to mzXML file format using MSConvert and were thereafter uploaded to the GNPS molecular networking server^4^ (Wang et al., 2016). On the GNPS platform, the MS/MS spectra were combined with the MSCluster algorithm, considering cosine similarity values (higher than 0.95) to create consensus MS spectra. For spectral networks, a parent mass and fragment ion tolerance of 0.5 and 0.2 Da were considered, respectively. For edge creation, cosine scores above 0.65, over four matched peaks, and at least two nodes in the top 10 cosine scores (K parameter) were fitted. The spectra were also searched against GNPS’s spectral libraries, considering scores above 0.65 and at least four matched peaks. The generated molecular network was imported and visualized as nodes and edges into Cytoscape 3.4.0 (Shannon et al., 2003). Nodes/spheres represent parent ions, and edge thickness corresponds to the cosine score between two nodes.

## Results and Discussion

### Phylogenetic Analysis and Taxonomic Identification of Actinobacteria Recovered From the Saint Peter and Saint Paul Archipelago and the Madeira Archipelago

All the BRA strains investigated in this study were identified as belonging to *Salinispora* based on the 16S rRNA gene similarity. The comparison of obtained sequences for BRA strains in the EzBioCloud and NCBI (BLAST) databases suggested a higher homology (over 99%) of most isolated strains with *S. arenicola*. The only exception was BRA-201, which presented a higher homology (over 99%) with *S. pacifica*. Interestingly, the opposite was observed for PTM strains, of which 33 of the 34 isolated strains were classified as *S. pacifica*, and only PTM-099 was classified as *S. arenicola* (Prieto-Davo et al., 2016).

**FIGURE 2:**
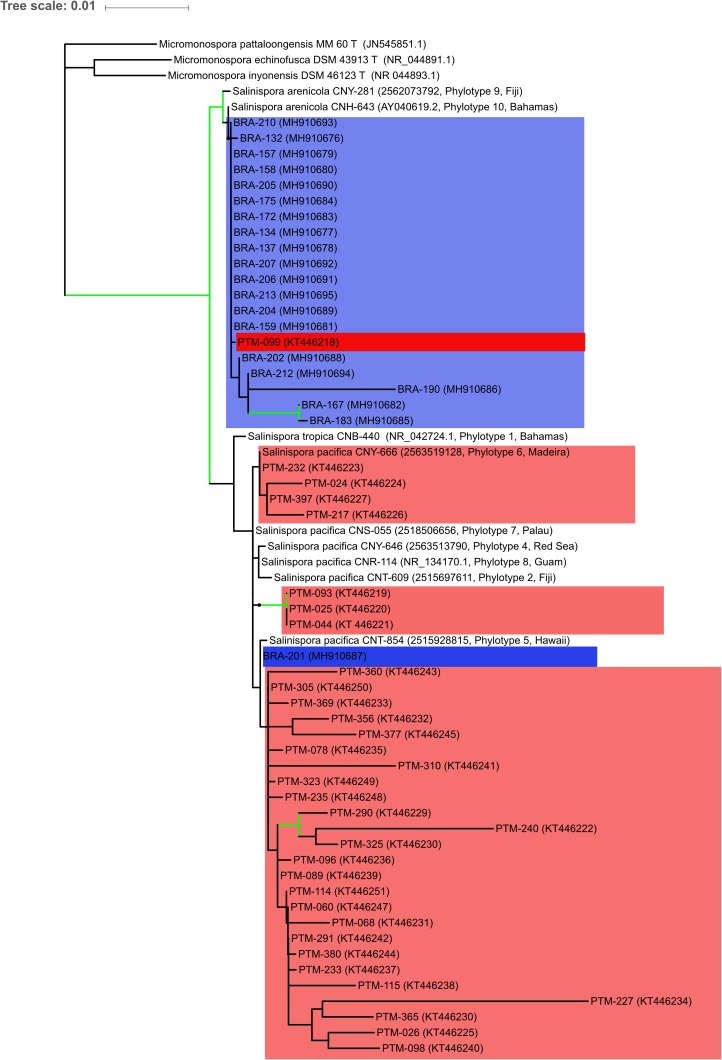
Maximum likelihood tree based on 16S rRNA gene sequences showing the phylogenetic relationships among the 53 actinobacteria from the genus *Salinispora* assessed in this study and 12 reference sequences, being 3 *Micromonospora* sequences to root the tree, and 9 *Salinispora* sequences representing phylotypes from Millan-Aguinaga et al. (2017). Blue color indicates the Brazilian strains, while red color indicates the Portuguese strains. Green lines mark nodes with > 70% bootstrap support. NCBI or IMG accession numbers are in brackets, along with Millan-Aguinaga et al. (2017) phylotype number and geographical location for reference sequences.

The identification was supported by a phylogenetic reconstruction using 16S rRNA data including sequences of *S. tropica, S. arenicola*, and *S. pacifica* from Millan-Aguinaga et al. (2017) (Figure 2). The tree shows that *S. arenicola* and *S. pacifica* are present in both oceanic islands, but the former seems to be more common in the Saint Peter and Saint Paul Archipelago, whereas most strains from the Madeira Archipelago are closely related to the latter species, as previously indicated by the EzBioCloud comparisons. *Salinispora arenicola* from the Atlantic Oceanic islands is more closely related to Millan-Aguinaga et al. (2017) phylotype 10, which is also from an Atlantic island. *Salinispora pacifica* is much more diverse, as previously shown by Millan-Aguinaga et al. (2017), which suggests that this species should be split in at least seven new species. The low bootstrap values within internal *S. pacifica* clades hinder further discussion regarding our strains’ assignment to any known phylotype. Nevertheless, the few supported clades are indicative of an even higher diversity for this species complex.

Both archipelagos are located in the North Atlantic Ocean, but they are over 2,370 nmi apart. The Saint Peter and Saint Paul Archipelago is located in the equatorial zone, whereas the Madeira Archipelago is located in the subtropical zone. There is also a striking difference between the two archipelagos in terms of size and oceanographic conditions (Supplementary Table S2). The Saint Peter and Saint Paul Archipelago is a very small set of nonvolcanic islets with few areas of sediment accumulation, because it is one of the rare cases in which mantle material produced the islets (Campos et al., 2010). The Madeira Archipelago, however, measures over 740 km^2^ and consists of three main islands, namely Madeira, Porto Santo, and Desertas, formed by typical hotspot volcanism. Studies of the presence of *Salinispora* in the Atlantic Ocean are scarce. For instance, Jensen et al. (2015) described the global distribution of this genus with records of 18 different locations, but only few of them were situated in the Atlantic. In the map shown in Figure 1A, we included the samples described in that study as well as four additional locations based on recent reports (Cosa and Okoh, 2012; Prieto-Davo et al., 2016; Ng and Tan, 2018; Yang and Song, 2018).

### Metabolic Profiling of *Salinispora* Strains

**FIGURE 3:**
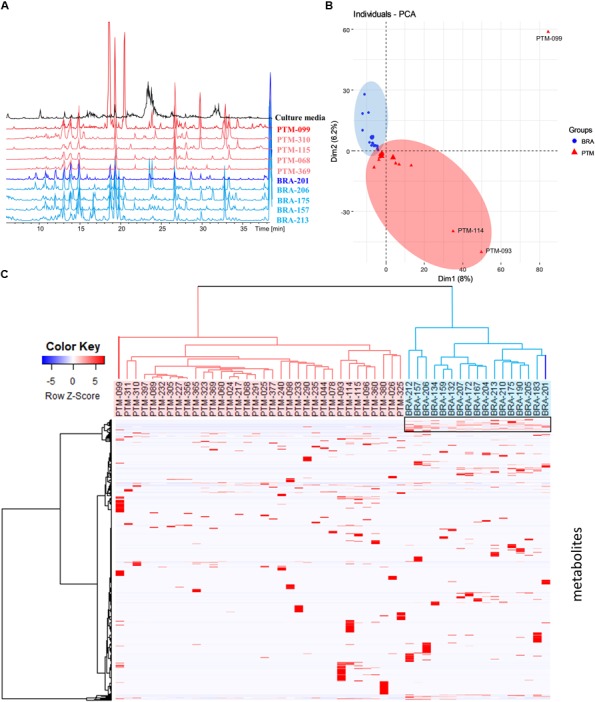
**(A)** Metabolic profiling (HPLC-MS) of some crude extracts produced by the *Salinispora* strains included in this study; **(B)** principal component analysis (PCA) was based on the table containing the peak area considering Pareto scaling; **(C)** hierarchical cluster analysis (HCA) was constructed pondering Euclidian distance and Ward’s method and showed on the top of the heatmap showing differential accumulation of mass features (right box) according to each group created in HCA.

To further characterize the isolated strains from the genus *Salinispora*, chromatographic profiling of their crude extracts was performed. Figure 3A shows the LC-MS profile of some representative samples. In the culture conditions considered here, the ethyl acetate extracts presented metabolites with distinct polarities, including a large group of higher polarity eluting between 10 and 16 min, composed mainly of diketopiperazines (Supplementary Figure S1) (Silva et al., 2017). Moreover, it is possible to clearly observe the presence of a greater number of chromatographic signals in the extracts produced by *S. arenicola* than *S. pacifica*. To better investigate the number of metabolites, these LC-MS data were processed using MZmine software. The data of BRA and PTM samples were processed together, including solvent blank and culture media controls. To avoid misinterpretation, the chromatographic data obtained for media controls and solvent blank were subsequently subtracted from the samples’ data. A total of 3,076 putative metabolites were detected, of which an average of 224 metabolites were produced by *S. arenicola* and 116 putative metabolites by *S. pacifica*, representing a ratio of approximately 2:1. These results are in agreement with the literature, which has described *S. arenicola* as a producer of a higher number of metabolites than *S. pacifica* strains (Bose et al., 2014; Duncan et al., 2015).

Metabolomic approaches employing MS and/or NMR data have been widely used in chemotaxonomy studies of plants and other organisms, including microorganisms. It is known that *Salinispora* species, even those sharing over 99% of 16S rRNA identity, produce species-specific metabolites, as noted above. However, Edlund et al. (2011) and Letzel et al. (2017) observed the presence of endemic genes, which underline the influence of the environment on the evolution of this genus. Nevertheless, the authors did not evaluate the expression of such genes at the metabolic level, which is crucial for comprehending the importance of such genes in *Salinispora* in terms of its environmental adaptation or even its chemical communication. Therefore, in order to observe trends of environmental influence on the metabolites’ production, the metabolic profiling was evaluated by multivariate analyses. Moreover, although the actinobacteria were recovered from different locations, they were cultured under the same conditions; thus, the table resulting from the MZmine processing, containing the peak area of the detected metabolites, was used for a comparative analysis. The PCA presented in Figure 3B shows that BRA and PTM samples are indeed metabolically similar. However, PTM-099, followed by PTM-093 and PTM-114, are distinct from all other samples. Even considering that most samples are chemically very close to each other, a trend of metabolic grouping by geographic origin could be observed. These trends are better visualized on HCA (Figure 3C), where the samples were separated into two distinct groups, one from Brazil (BRA, blue) and the other from Portugal (PTM, red). Particularly in the latter, PTM-099 is the most different; it is the only *S. arenicola* of the PTM group. These results show that even though the actinobacteria were cultured under the same conditions and are capable of producing species-specific metabolites, the environmental influence on their metabolic expression is evident. In particular, despite BRA-201 being the only *S. pacifica* of the BRA group, its metabolic profile is more related to all other Brazilian strains, which are *S. arenicola*, and the same metabolic pattern was observed for PTM-099, which, despite being *S. arenicola*, has a metabolic profile chemically related to all the other Portuguese strains, identified as *S. pacifica*. These findings point to the presence of endemic gene clusters, possibly shared locally through horizontal gene transfer, as a result of which the actinobacteria express a geographic-specific metabolic profile.

In view of these results, we then investigated the metabolites responsible for these grouping trends. Hence, a heatmap (Figure 3C) was constructed according to the grouping observed in the HCA. As shown in Figure 3C, highlighted in a black box, a group of chemical compounds is present in all BRA *Salinispora*. These compounds may be responsible for grouping the BRA samples separately from PTM samples, given that these compounds are absent in all PTM samples. To achieve better visualization, we have presented these results in a heatmap; seemingly, the occurrence of minor metabolites has led to the separation of samples by geographic origin.

### Comparison of Chemical Diversity Produced by *Salinispora* BRA and PTM

**FIGURE 4:**
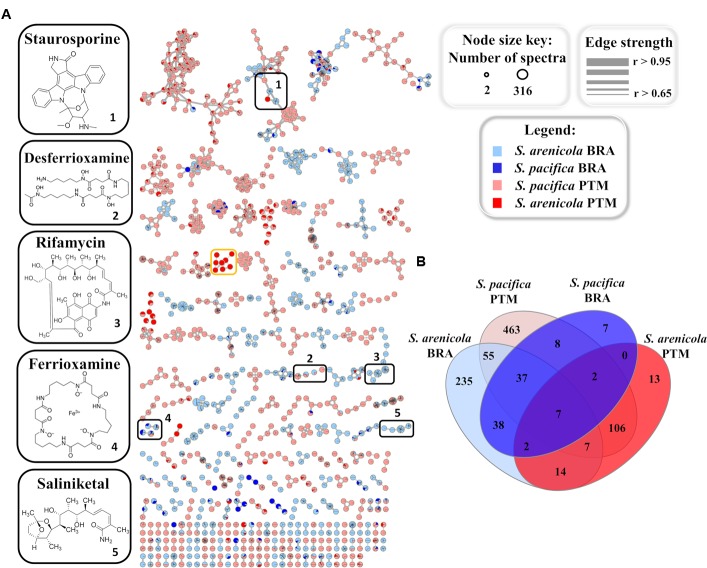
**(A)** Molecular networking of *Salinispora* BRA and PTM recovered from the Atlantic Ocean, considering the positive ionization mode (ESI+) data, after removal of media controls and solvent blanks. Nodes represent parent ions, according to the origin of the actinobacteria strain, and edge thickness corresponds to the cosine score, which represents the degree of similarity between the connected nodes. Only clusters containing at least two nodes are shown. **(B)** Venn diagram with the parent ions present in the molecular network generated for *Salinispora* BRA and PTM strains.

To further investigate and compare the metabolic profile produced by the Brazilian and Portuguese *Salinispora* strains, all crude extracts were subjected to an untargeted method, which fragments the three most intense ions detected in each scan in the LC-MS analyses, resulting in the acquisition of LC-MS/MS data. This approach yields a data set more representative of the total chemical composition of the extracts. The acquired MS/MS data were used to create a molecular network on the online GNPS platform^5^ (Wang et al., 2016). Molecules from the same chemical class or group with similar structures usually exhibit similar fragmentation patterns. Therefore, this online molecular tool is able to compare each MS/MS spectrum within the entire experimental data set, connecting spectra with an edge/line with greater statistical similarity, thus arranging them into groups, and separating groups that exhibit different fragmentation patterns. In order to improve the quality and reliability of this analysis, culture media blanks obtained by the extraction of culture media with no actinobacteria were used. Figure 4A shows the generated molecular network, after media control and solvent blank removal, in which each node/sphere represents a parent ion of an MS/MS spectrum. The color represents the origin of the actinobacteria strain according to the legend.

The resulting molecular network exhibited 994 nodes organized in 219 clusters of at least two parent ions. Of these, 280 parent ions were detected exclusively in BRA samples, 582 were found exclusively in PTM samples and 132 were shared by both. It is important to point out that the molecular network represents an overview of the chemical groups and how they are distributed among the samples. The number of parent ions (nodes) in the network does not necessarily represent the number of metabolites present in the samples, because mass spectrometry can generate different charge states and/or adducts with different ions (H^+^, Na^+^, K^+^, and NH_3_^+^, among others) for the same molecule. Moreover, poor-quality MS/MS spectra, which might result from parent ions at low intensity, were not considered in the construction of this molecular network, which explains the smaller number of parent ions in relation to the number of metabolites previously discussed.

Regarding the molecular families present in the network (Figure 4A), approximately 40% overlapped with BRA and PTM samples, and around 22% were produced solely by BRA *Salinispora* strains, which exhibited higher similarity among each other. Nine molecular families in the network were found to be species-specific, regardless of their geographic location. Seven of them were produced only by *S. arenicola*, including BRA and PTM samples, whereas the other two were produced by *S. pacifica* strains, both from BRA and PTM samples. Interestingly, one molecular family of eight metabolites, with molecular masses around 360 and 480 a.m.u., was found exclusively in *S. pacifica* PTM-099 (orange box in Figure 4A, detailed in Supplementary Figure S2), highlighting this actinobacterium as a unique and interesting strain that deserves to be studied in-depth in future research. Considering the species, independently of their geographic origin, around 22% of the molecular families were produced exclusively by *S. arenicola* and around 30% by *S. pacifica*, and the remaining metabolites were found to be shared by both species. The Venn diagram presented in Figure 4B shows that at the molecular level, 48% of these metabolites were produced exclusively by *S. pacifica* and 26% exclusively by *S. arenicola*.

### Known Molecular Families in the Network Produced by *Salinispora* BRA and PTM Strains

GNPS also has a natural product library of MS/MS spectra of previously reported metabolites. When a spectrum acquired for a given sample is highly similar (that is, a match) with an MS/MS spectrum from the GNPS library, it is recognized and annotated. Most molecular families in the network were not annotated by the GNPS library, which means that the metabolites produced by the *Salinispora* strains investigated in this study have not yet been deposited in the library or are novel metabolites. Because most of the known molecules produced by the obligate marine genus *Salinispora* are in the GNPS library, we presume that several new chemical structures are being produced by the strains investigated here. Regarding the known molecular families, this approach enabled the identification of five different chemical classes in the crude extracts: the anticancer agents staurosporines and saliniketals, the antibiotic rifamycin, the metal complexing agents (siderophore) ferrioxamines and desferrioxamines, and diketopiperazines. All these chemical classes, except the signaling diketopiperazines, are highlighted in black boxes in Figure 4A. The information regarding the distribution of the ions that compose the highlighted molecular families are summarized in Table 1. In general, BRA strains were much more representative of the identified molecular families, producing at least one compound from each. Even BRA *S. pacifica*, the only different species from Brazilian strains, produced ferrioxamine. On the other hand, Portuguese strains were less representative; only a few PTM strains were able to produce some staurosporine, ferrioxamine and desferrioxamine derivatives. In particular, PTM-093, PTM-114, PTM-360, and PTM-365 stand out for producing more than one compound from these families.

**Table 1:** Metabolite profiles of all 54 *Salinispora* strains isolated from sediments from the Saint Peter and Saint Paul and Madeira Archipelagos, listing the compounds identified by the GNPS library.

Clusters																				
	Saliniketal				Rifamycin				Staurosporine			Ferrioxamine				Desferrioxamine			
Strains	418.429	432.385	434.255	434.361	718.587	736.494	748.585	750.556	467.448	481.573	483.434	611.538	625.572	640.502	654.520	593.521	595.566	607.537	623.552
BRA-132	X	X			X	X	X	X				X	X	X	X				X
BRA-134	X	X			X	X	X	X	X		X								
BRA-137	X																		
BRA-157	X	X			X		X	X											
BRA-158	X	X																	
BRA-159	X				X			X	X		X								
BRA-167	X			X	X			X	X		X				X				
BRA-172	X				X			X	X		X								X
BRA-175	X			X	X			X					X						
BRA-183	X				X			X	X		X		X						X
BRA-190	X				X			X	X		X								
BRA-201												X	X	X	X				
BRA-202	X																		
BRA-204	X	X			X			X	X		X		X			X		X	
BRA-205	X	X			X			X	X		X		X		X				X
BRA-206	X				X		X	X	X			X	X	X	X			X	
BRA-207	X	X			X			X					X		X				
BRA-210	X				X			X	X		X								
BRA-212	X		X		X								X						X
BRA-213	X	X			X			X	X		X								
PTM-024																			
PTM-025																			
PTM-026																			
PTM-044																			
PTM-060																			
PTM-068																			
PTM-078										X									
PTM-089																			
PTM-093															X				X
PTM-096																		X	
PTM-098															X				
PTM-099	X				X														
PTM-114													X		X			X	X
PTM-115																			
PTM-217																			
PTM-227															X				
PTM-232																			
PTM-235																			
PTM-240																			
PTM-290										X									
PTM-291																			
PTM-305											X								X
PTM-310																			
PTM-311																			
PTM-323																			
PTM-325																			
PTM-356																			
PTM-360													X	X	X	X	X	X	X
PTM-365															X			X	X
PTM-369																			
PTM-377																			
PTM-380																			
PTM-397																			

*Light Blue, Strains identified as *Salinispora arenicola* based on 16S rRNA sequencing collected in Saint Peter and Saint Paul’s Archipelago; Dark blue, Strain identified as *Salinispora pacifica* based on 16S rRNA sequencing collected in Saint Peter and Saint Paul’s Archipelago; Light Red, Strains identified as *Salinispora pacifica* based on 16S rRNA sequencing collected in Madeira Archipelago; Dark Red, Strain identified as *Salinispora arenicola* based on 16S rRNA sequencing collected in Madeira Archipelago*.

The hydroxamate siderophores ferrioxamine and desferrioxamine were produced by both BRA *S. arenicola* and PTM *S. pacifica* strains (Supplementary Figure S3), and the later chemical class was also produced by the BRA *S. pacifica*. In total, this molecular family was detected in approximately 30% of the investigated strains. Interestingly, the desferrioxamine family was recently detected in extracts produced by *Salinispora* strains, when Crusemann et al. (2017) employed molecular network at the GNPS. Previously, desferrioxaminse had been described only for *Streptomyces* strains.

Regarding the staurosporine family (Supplementary Figure S4), staurosporine itself was detected in 20% of the samples from BRA *S. arenicola* strains. However, there are other staurosporine derivatives. Hydroxy-staurosporine, for instance, was also detected in 20% of the samples, being mainly produced by BRA *S. arenicola*, but also by one PTM *S. pacifica*. The other identified derivative of this family, oxo-staurosporine, was found only in PTM *S. pacifica* strains (4% of the samples). This data shows that this chemical class is produced by both *Salinispora* species.

**FIGURE 5:**
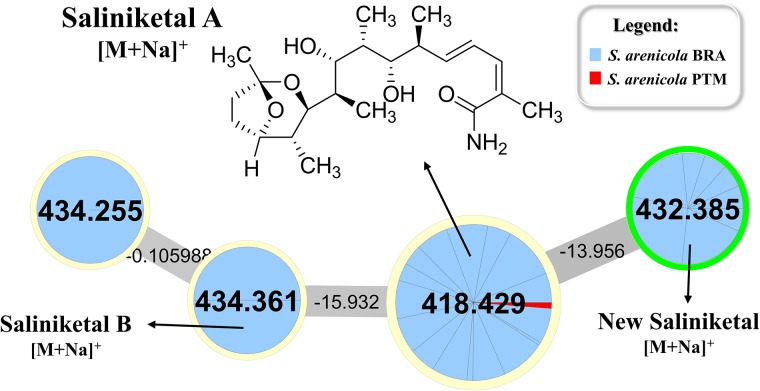
Cluster for saliniketals ([M+Na]^+^) produced by *S. arenicola* BRA and PTM. Nodes with *m/z* 418 and *m/z* 434 refer to saliniketals A and B, respectively, as indicated. The second node, with *m/z* 434, refers to an isomer of saliniketal B, whereas the node with *m/z* 432 is a derivative of saliniketal A with one additional –CH_2_.

Rifamycin and the unusual bicyclic polyketides saliniketal A and B were produced exclusively by *S. arenicola*, including the PTM strain (Supplementary Figure S5 and Figure 5, respectively), as described by Jensen et al. (2007) and Jensen et al. (2015). The presence of the saliniketal in all *S. arenicola* strains may explain the anticancer results against human colon carcinoma cell line HCT-116, previously reported for BRA and PTM extracts (Ferreira et al., 2016; Prieto-Davo et al., 2016), which revealed activity for *S. arenicola* and no activity for *S. pacifica*, as compiled in Supplementary Table S1. Despite being produced here only by *S. arenicola* strains, rifamycins (Supplementary Figure S5) are also produced by other bacteria genera, such as *Amycolatopsis* (Krishna et al., 1998) and *Streptomyces* (Sensi et al., 1959), whereas saliniketal has been described in the literature solely for *S. arenicola*. The cluster from the network presented in Figure 5 shows the presence of saliniketal A (*m/z* 418) and B (*m/z* 434) and two other saliniketal derivatives, one isomer of saliniketal B (*m/z* 434) and a putative new saliniketal (*m/z* 432), which refers to a derivative of saliniketal A with one additional –CH_2_, found in 38% of the BRA *S. arenicola* strains. Of these derivatives, only PTM *S. arenicola* strain reveals the production of saliniketal A. Because saliniketals are promising anticancer compounds (Williams et al., 2007; Olano et al., 2009), the discovery of new derivatives of this chemical class represents an interesting finding in this context. It is important to emphasize that rifamycin S and saliniketal A were produced by 100% of the *S. arenicola* strains investigated in this study, suggesting that the gene cluster of this biosynthetic pathway is highly conserved in this species. Therefore, considering that rifamycin derivatives are also produced by other genera, saliniketal A might be considered a chemical marker for *S. arenicola*.

This study demonstrated that the majority of *Salinispora* strains recovered from sediments from the Saint Peter and Saint Paul Archipelago belongs to the species *S. arenicola* and that the preponderance of strains recovered from the Madeira Archipelago belongs to *S. pacifica*. By using metabolomics tools, applied mainly to the investigation of secondary metabolites, we provided further support for the occurrence of some typical species-specific chemical markers among the studied strains. However, these analyses have broadened the notion that environmental factors strongly affect the secondary metabolism of *Salinispora* species, thus revealing that geography plays a stronger role than pure taxonomy in the already prolific natural product chemistry of this genus of actinobacteria.

## Author Contributions

All authors listed have made a substantial, direct and intellectual contribution to the work, and approved it for publication.

## Conflict of Interest Statement

The authors declare that the research was conducted in the absence of any commercial or financial relationships that could be construed as a potential conflict of interest.

## Footnotes

1 http://www.ncbi.nlm.nih.gov/genbank

2 https://www.ezbiocloud.net/

3 https://img.jgi.doe.gov

4 gnps.ucsd.edu

5 http://gnps.ucsd.edu
