# Supplementary material for: Metabolomic Fingerprinting of Salinispora From Atlantic Oceanic Islands
Source: Front Microbiol. 2018 Dec 11;9:3021. doi: 10.3389/fmicb.2018.03021 (PMC6297358; doi:10.3389/fmicb.2018.03021)

**SUPPLEMENTARY INFORMATION**

**Metabolomic fingerprinting of *Salinispora* from Atlantic Oceanic islands**

Anelize Bauermeister^1,2^, Karen Velasco-Alzate^1^, Tiago Dias^3^, Helena Macedo^3^, Elthon G. Ferreira^4^, Paula C. Jimenez^1,5^, Tito M. C. Lotufo^6^, Norberto P. Lopes^2^, Susana P. Gaudêncio^3*^, Letícia V. Costa-Lotufo^1*^

^1^ Departamento de Farmacologia, Instituto de Ciências Biomédicas, Universidade de São Paulo, São Paulo/SP, Brasil

^2^ Núcleo de Pesquisa em Produtos Naturais e Sintéticos (NPPNS), Faculdade de Ciências Farmacêuticas de Ribeirão Preto, Universidade de São Paulo, Ribeirão Preto/SP, Brasil

^3^ UCIBIO-REQUIMTE, Departamento de Química e Departamento de Ciências da Vida, Laboratório de Biotecnologia Azul e Biomedicina, Faculdade de Ciências e Tecnologia, Universidade NOVA de Lisboa, 2829-516 Caparica, Portugal

^4^ Departamento de Química Orgânica e Inorgânica, Universidade Federal do Ceará, Fortaleza, CE, 60451-970, Brasil

^5^ Departamento de Ciências do Mar, Universidade Federal de São Paulo, Santos, SP, 11.070-100, Brasil

^6^ Instituto Oceanográfico, Universidade de São Paulo, São Paulo, SP, 05508-120, Brasil

*Corresponding authors: Letícia Veras Costa-Lotufo (phone: +55-11-30917316; e-mail: [costalotufo@usp.br](mailto:costalotufo@usp.br)) and Susana P. Gaudêncio (phone: +351-21-2948300; e-mail: [s.gaudencio@fct.unl.pt](mailto:s.gaudencio@fct.unl.pt))

**Table S1**. *Salinispora* strains isolated from sediments from the Saint Peter and Saint Paul Archipelago and Madeira Archipelago, and the compilation of their anticancer activity results against human colon carcinoma cell line HCT-116 (Ferreira et al, 2016; Prieto-Davo at al., 2016).

| **Strain** | **Site** | **Latitude (N)** | **Longitude (W)** | **Depth (m)** | **Date** | **Method** | **Media** | **HCT-116**  **IC_50_**  **(μg/mL)** |
| --- | --- | --- | --- | --- | --- | --- | --- | --- |
| **BRA-132** | B2 | 0° 55' 1.6824'' | 29° 20' 46.1364'' | 35 | Feb 19th, 2011 | M1 | SWA | 15.07 |
| **BRA-134** | B2 | 0° 55' 1.6824'' | 29° 20' 46.1364'' | 35 | Feb 19th, 2011 | M1 | TM | 11.13 |
| **BRA-137** | B3 | 0° 55' 2.3556'' | 29° 20' 44.3976'' | 8 | Feb 20th, 2011 | M2 | SWA | 16.06 |
| **BRA-157** | B2 | 0° 55' 1.6824'' | 29° 20' 46.1364'' | 35 | Feb 19th, 2011 | M1 | SWA | 1.14 |
| **BRA-158** | B2 | 0° 55' 1.6824'' | 29° 20' 46.1364'' | 35 | Feb 19th, 2011 | M1 | SWA | nt |
| **BRA-159** | B2 | 0° 55' 1.6824'' | 29° 20' 46.1364'' | 35 | Feb 19th, 2011 | M1 | SWA | 0.80 |
| **BRA-167** | B1 | 0° 54' 58.6476'' | 29° 20' 47.6412'' | 33 | Feb 19th, 2011 | M1 | SCA | 0.15 |
| **BRA-172** | B2 | 0° 55' 1.6824'' | 29° 20' 46.1364'' | 35 | Feb 19th, 2011 | M1 | TM | 0.14 |
| **BRA-175** | B2 | 0° 55' 1.6824'' | 29° 20' 46.1364'' | 35 | Feb 19th, 2011 | M1 | SWA | 0.41 |
| **BRA-183** | B3 | 0° 55' 2.3556'' | 29° 20' 44.3976'' | 8 | Feb 20th, 2011 | M1 | SWA | 1.65 |
| **BRA-190** | B2 | 0° 55' 1.6824'' | 29° 20' 46.1364'' | 35 | Feb 19th, 2011 | M1 | SWA | 0.12 |
| **BRA-201** | B1 | 0° 54' 58.6476'' | 29° 20' 47.6412'' | 33 | Feb 19th, 2011 | M1 | SWA | > 50 |
| **BRA-202** | B2 | 0° 55' 1.6824'' | 29° 20' 46.1364'' | 35 | Feb 19th, 2011 | M1 | TM | 2.19 |
| **BRA-204** | B2 | 0° 55' 1.6824'' | 29° 20' 46.1364'' | 35 | Feb 19th, 2011 | M1 | SWA | 0.70 |
| **BRA-205** | B2 | 0° 55' 1.6824'' | 29° 20' 46.1364'' | 35 | Feb 19th, 2011 | M1 | TM | 0.71 |
| **BRA-206** | B1 | 0° 54' 58.6476'' | 29° 20' 47.6412'' | 33 | Feb 19th, 2011 | M1 | SWA | 3.55 |
| **BRA-207** | B2 | 0° 55' 1.6824'' | 29° 20' 46.1364'' | 35 | Feb 19th, 2011 | M1 | SWA | 0.25 |
| **BRA-210** | B2 | 0° 55' 1.6824'' | 29° 20' 46.1364'' | 35 | Feb 19th, 2011 | M1 | SWA | 0.21 |
| **BRA-212** | B2 | 0° 55' 1.6824'' | 29° 20' 46.1364'' | 35 | Feb 19th, 2011 | M1 | TM | > 50 |
| **BRA-213** | B2 | 0° 55' 1.6824'' | 29° 20' 46.1364'' | 35 | Feb 19th, 2011 | M1 | TM | 0.07 |
| **PTM-024** | C2 | 32° 59.892’ | 16° 22.956’ | 12 | Jun 10th, 2012 | M2 | SWA | > 250 |
| **PTM-025** | C6 | 33° 01.785’ | 16° 23.118’ | 12 | Jun 10th, 2012 | M2 | SWA | >250 |
| **PTM-026** | C2 | 32° 43.901’ | 17° 12.488’ | 13 | Jun 12th, 2012 | M2 | SWA | >250 |
| **PTM-044** | C6 | 32° 59.892’ | 16° 22.956’ | 12 | Jun 10th, 2012 | M2 | SWA | >250 |
| **PTM-060** | C6 | 33° 00.216’ | 16° 22.792’ | 16 | Jun 10th, 2012 | M1 | 1/2A1 | >250 |
| **PTM-068** | C12 | 32° 31.686’ | 16° 31.241’ | 10 | Jun 14th, 2012 | M1 | SWA | >250 |
| **PTM-078** | C9 | 33° 03.155’ | 16° 16.700’ | 16 | Jun 08th, 2012 | M1 | 1/2A1 | >250 |
| **PTM-089** | C6 | 32° 59.892’ | 16° 22.956’ | 12 | Jun 10th, 2012 | M1 | 1/2A1 | >250 |
| **PTM-093** | C6 | 32° 59.892’ | 16° 22.956’ | 12 | Jun 10th, 2012 | M2 | SWA | >250 |
| **PTM-096** | C10 | 32° 35.346’ | 16° 33.060’ | 15 | Jun 14th, 2012 | M1 | SWA | >250 |
| **PTM-098** | C10 | 32° 34.655’ | 16° 32.648’ | 15 | Jun 14th, 2012 | M2 | SWA | >250 |
| **PTM-099** | C10 | 32° 35.346’ | 16° 33.060’ | 15 | Jun 14th, 2012 | M2 | SWA | 4.94 |
| **PTM-114** | C4 | 32° 40.200’ | 16° 48.072’ | 16 | Jun 08th, 2012 | M2 | SWA | >250 |
| **PTM-115** | C12 | 32° 32.193’ | 16° 31.971’ | 12 | Jun 14th, 2012 | M2 | A1 | >250 |
| **PTM-217** | C2 | 32° 43.901’ | 17° 12.488’ | 13 | Jun 12th, 2012 | M2 | SWA | >250 |
| **PTM-227** | C5 | 32° 43.680’ | 16° 44.948’ | 20 | Jun 08th, 2012 | M2 | SWA | >250 |
| **PTM-232** | C1 | 32° 46.958’ | 17° 14.790’ | 12 | Jun 12th, 2012 | M2 | SWA | >250 |
| **PTM-235** | C13 | 32° 30.713’ | 16° 30.535’ | 12 | Jun 14th, 2012 | M2 | SWA | >250 |
| **PTM-240** | C9 | 33° 03.155’ | 16° 16.700’ | 16 | Jun 09th, 2012 | M2 | SWA | >250 |
| **PTM-290** | C4 | 32° 40.200’ | 16° 48.072’ | 16 | Jun 08th, 2012 | M2 | SWA | >250 |
| **PTM-291** | C12 | 32° 32.193’ | 16° 31.971’ | 12 | Jun 14th, 2012 | M2 | SWA | >250 |
| **PTM-305** | C8 | 33° 03.180’ | 16° 17.080’ | 10 | Jun 09th, 2012 | M2 | SWA | >250 |
| **PTM-310** | C5 | 32° 44.509’ | 16° 41.839’ | 17 | Jun 08th, 2012 | M1 | A1 | >250 |
| **PTM-311** | C11 | 32° 32.193’ | 16° 31.971’ | 12 | Jun 14th, 2012 | M1 | 1/2A1 | >250 |
| **PTM-323** | C13 | 32° 30.713’ | 16° 30.535’ | 12 | Jun 14th, 2012 | M1 | 1/2A1 | >250 |
| **PTM-325** | C11 | 32° 31.686’ | 16° 31.241’ | 10 | Jun 14th, 2012 | M2 | SWA | >250 |
| **PTM-356** | C12 | 32° 30.713’ | 16° 30.535’ | 12 | Jun 14th, 2012 | M2 | SWA | >250 |
| **PTM-360** | C3 | 32° 42.364’ | 17° 09.071’ | 14 | Jun 12th, 2012 | M2 | SWA | >250 |
| **PTM-365** | C3 | 32° 42.364’ | 17° 09.071’ | 14 | Jun 12th, 2012 | M2 | SWA | >250 |
| **PTM-369** | C13 | 32° 31.686’ | 16° 31.241’ | 10 | Jun 14th, 2012 | M2 | SWA | >250 |
| **PTM-377** | C1 | 32° 46.958’ | 17° 14.790’ | 12 | Jun 12th, 2012 | M2 | SWA | >250 |
| **PTM-380** | C6 | 32° 59.892’ | 16° 22.956’ | 12 | Jun 10th, 2012 | M1 | 1/2A1 | >250 |
| **PTM-397** | C11 | 32° 32.193’ | 16° 31.971’ | 12 | Jun 14th, 2012 | M2 | SWA | >250 |

***TMA:** Artificial seawater (28.65 g/L of salt from the Red Sea) mixed with: glicose, yeast extract, K_2_HPO_4_, Na_2_HPO_4_, KNO_3_, NaCl, MgSO_4_.7H_2_O and CaCl_2_.2H_2_O, and a solution of trace metals (FeSO_4_.7H_2_O, ZnSO_4_.7H_2_O, MnSO_4_.4H_2_O, CuSO_4_.5H_2_O, CoSO_4_.7H_2_O, H_3_BO_3_, (NH_4_) 6Mo_7_O_24_.4H_2_O), diluted with distilled water; **SWA:** artificial seawater (28.65 g/L of salt from the Red Sea) and agar; **SCA**: starch, casein, agar; **A1:** artificial seawater (28.65 g/L of salt from the Red Sea), starch, peptone, yeast extract and agar; **1/2A1**: A1 diluted by 2; M1: dry sediment stamped into the plates with medium; M2: 50 µL of sterile SWA containing the sediment inoculated into the plates with medium. n.t. – not tested.

**Table S2**. Environmental data from São Pedro and São Paulo Archipelago (ASPSP) and Madeira Islands obtained from Bio-Oracle v.2*. SST = Sea Surface Temperature; Diss. Oxyg. = Dissolved Molecular Oxygen.

| Location | SST max (°C) | SST min (°C) | SST mean (°C) | Salinity | Diss. Oxyg. (mol.m-3) | Nitrate (mol.m-3) | Mean Chlorophyll (mg.m-3) | Calcite (mol.m-3) | pH | Phosphate (mol.m-3) |
| --- | --- | --- | --- | --- | --- | --- | --- | --- | --- | --- |
| ASPSP | 28.480 | 26.997 | 27.776 | 35.829 | 4.646 | 0.559 | 0.00438 | 6.4e-05 | 8.221 | 0.0680 |
| Madeira | 23.621 | 17.715 | 20.362 | 36.714 | 5.244 | 0.727 | 0.03860 | 6.3e-05 | 8.234 | 0.0435 |

*Assis, J., Tyberghein, L., Bosh, S., Verbruggen, H., Serrão, E. A., & De Clerck, O. (2018). Bio-ORACLE v2.0: Extending marine data layers for bioclimatic modelling. Global Ecology and Biogeography, 27(3): 277-284. https://doi.org/10.1111/geb.12693

**Figure S1.** MS/MS spectra of diketopiperazines produced by *Salinispora* strains.

**
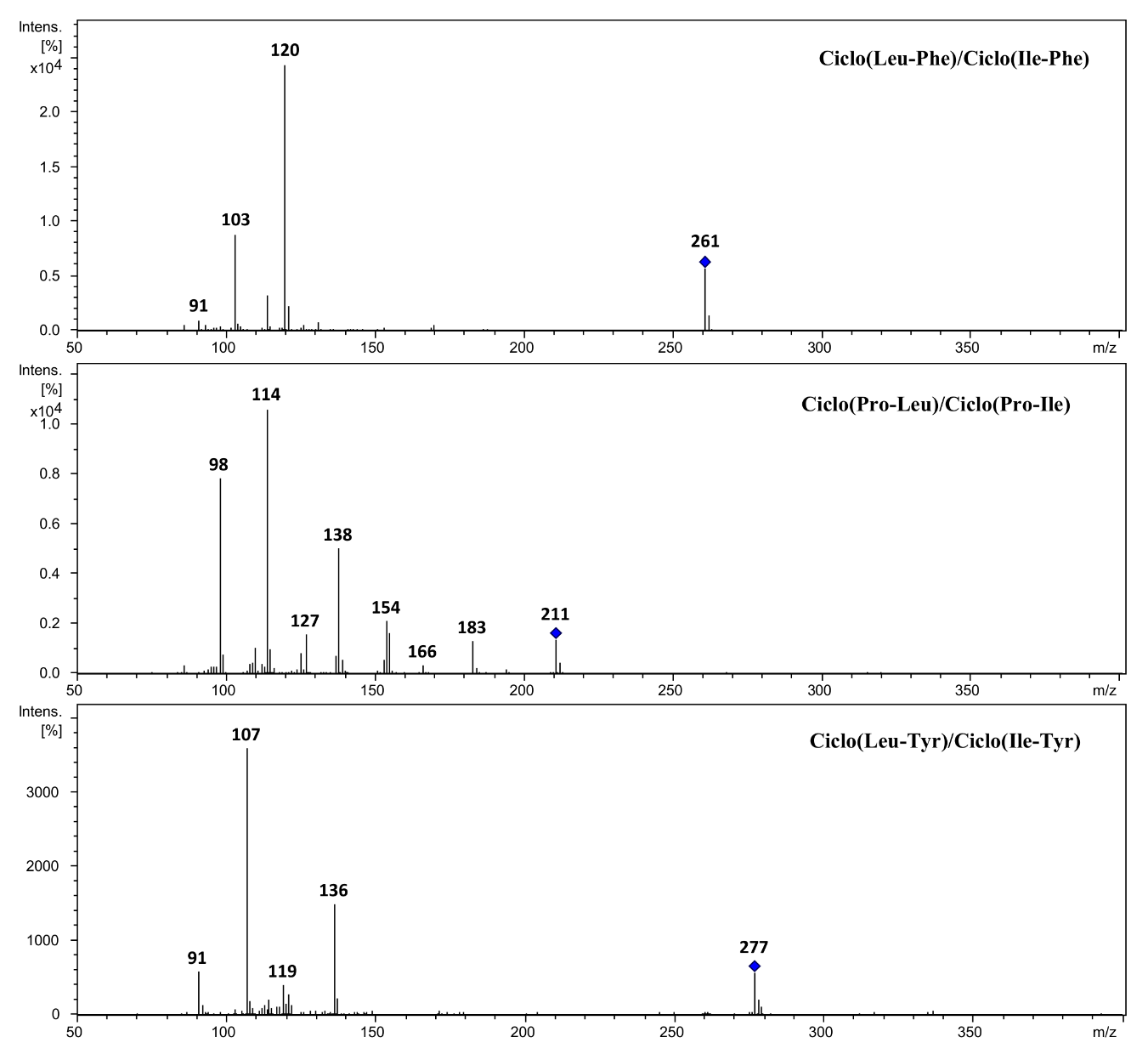
**

**Figure S2.** Cluster of metabolites produced exclusively by *S. arenicola* PTM-099. The edge strength is according to the cosine scores.

**
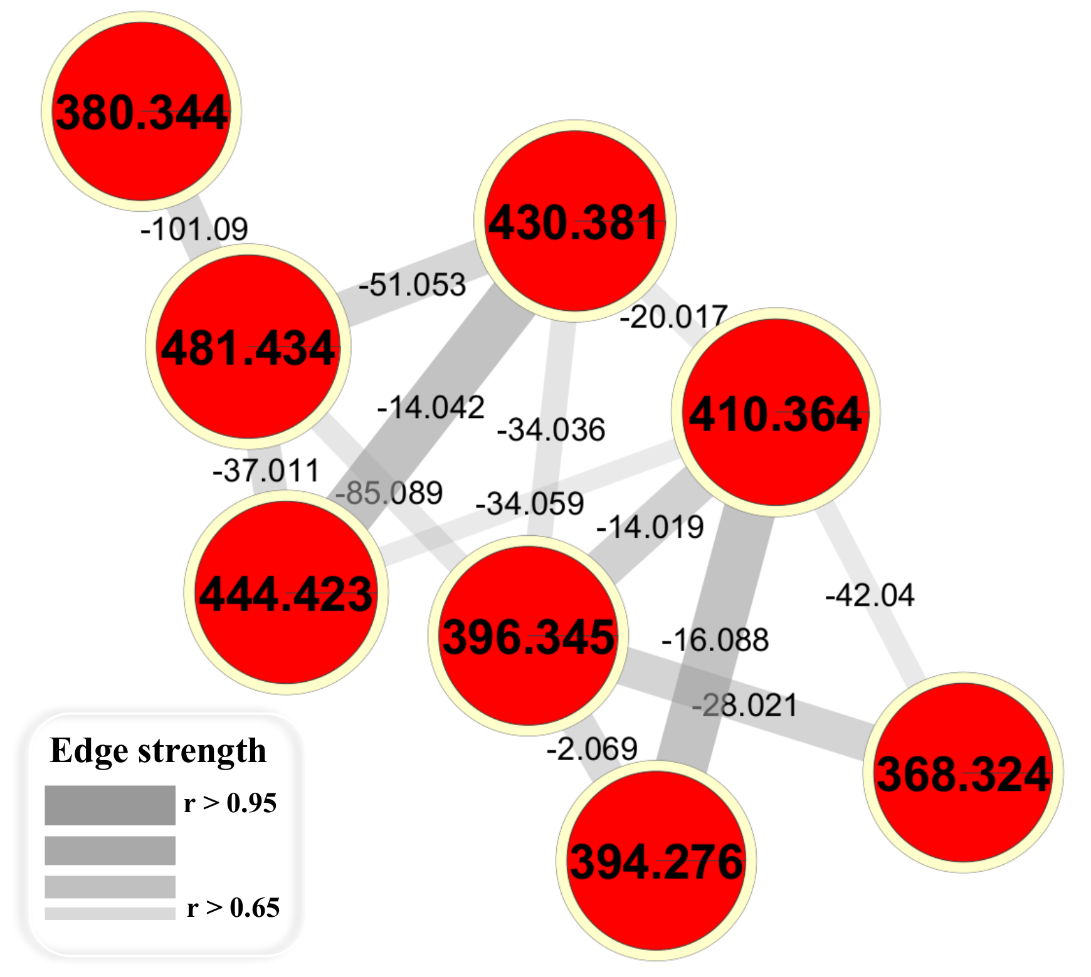
**

**Figure S3.** Cluster of (A) desferrioxamine and (B) ferrioxamine families observed as sodiated adducts ([M+Na]^+^) produced by BRA and PTM *Salinispora* strains. The node color represents the origin of the actinobacteria, and edge strength is according to the cosine scores.


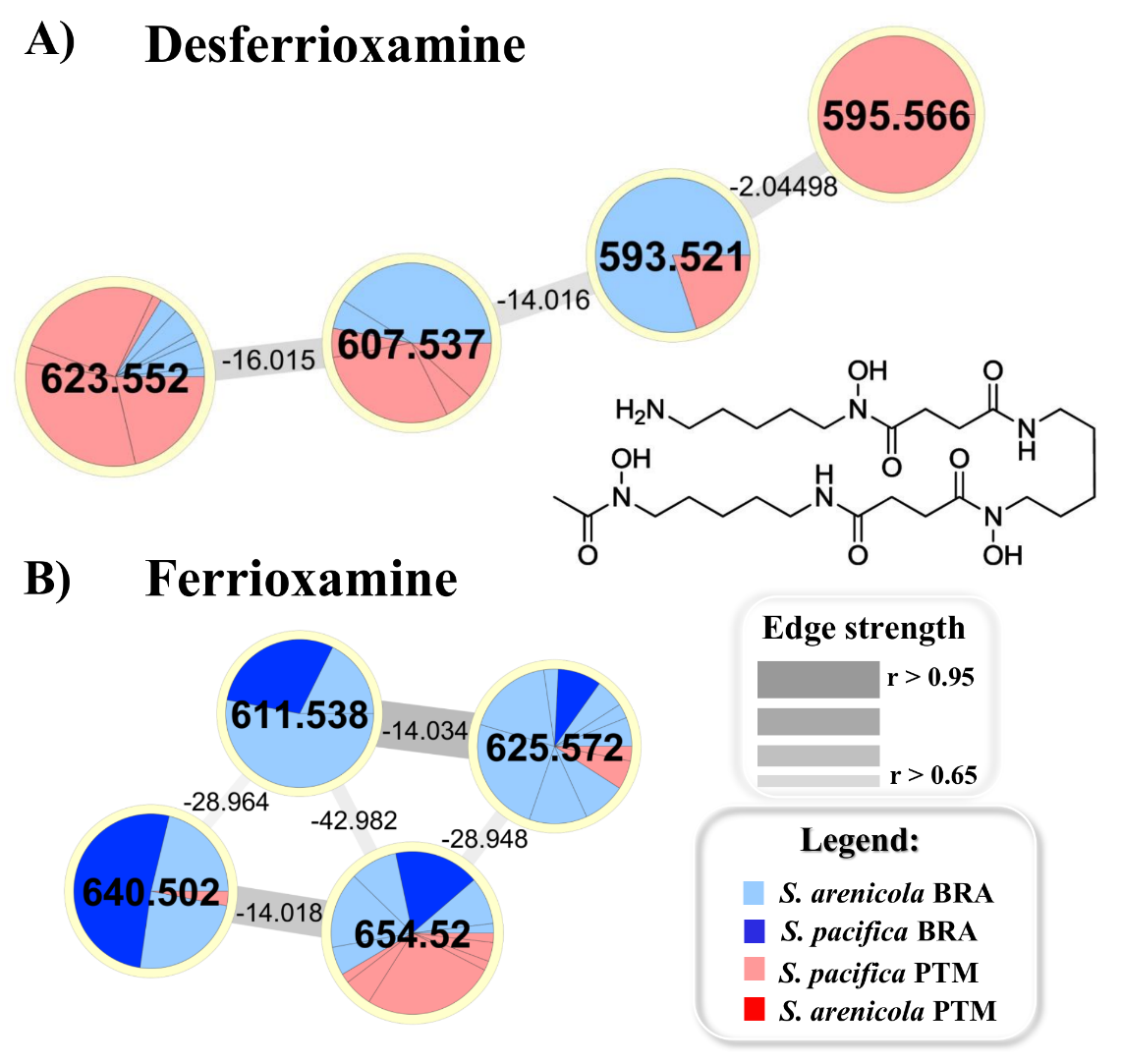


**Figure S4.** Cluster of the staurosporine family observed as protonated molecules ([M+H]^+^) produced by BRA and PTM *Salinispora* strains. The node color represents the origin of the actinobacteria, and edge strength is according to the cosine scores.


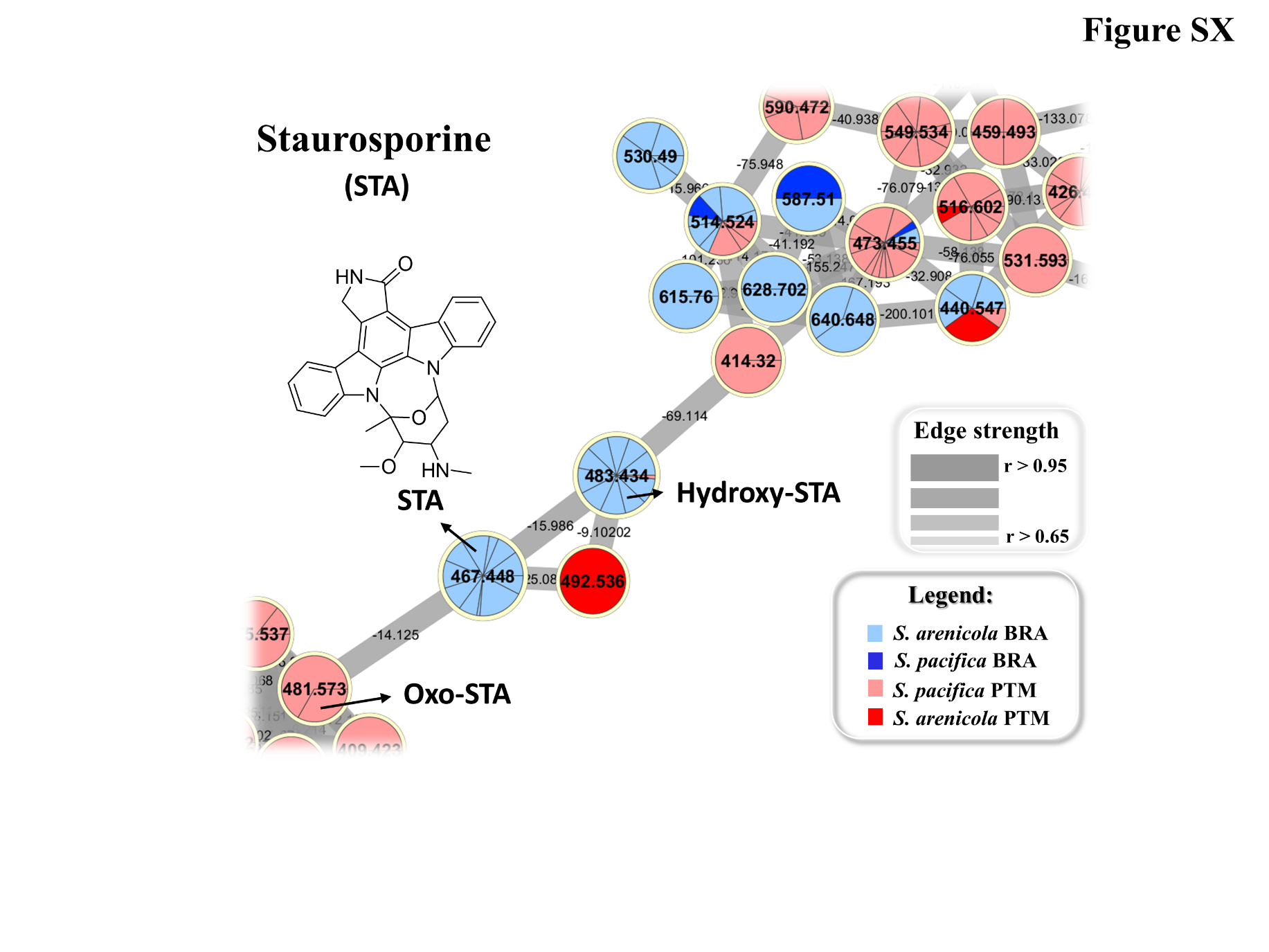


**Figure S5.** Cluster of the rifamycin family observed as sodiated adducts ([M+Na]^+^) produced by BRA and PTM *S. arenicola* strains. The node with *m/z* 718 refers to rifamycin S. The node color represents the origin of the actinobacteria, and edge strength is according to the cosine scores.


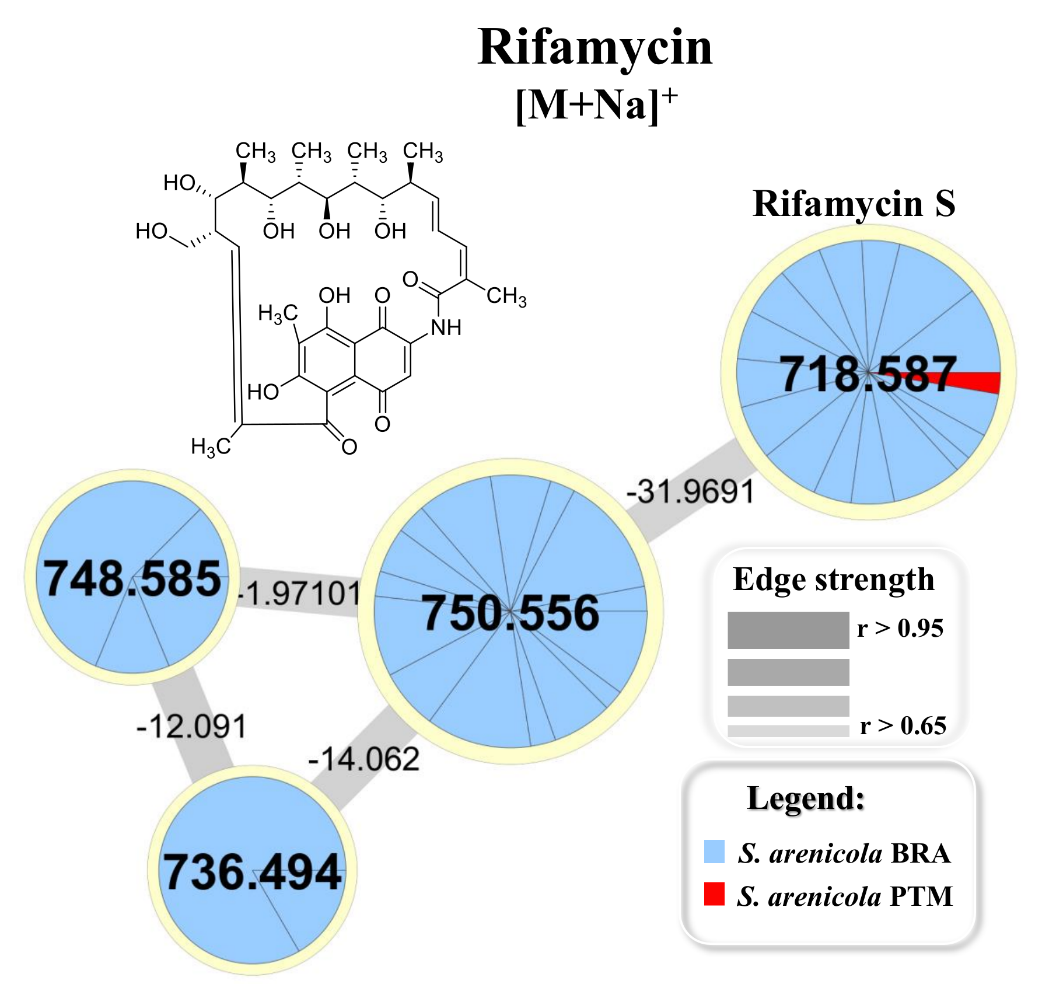

Supplement: Supplementary file 1 [file Data_Sheet_1.docx]
